# Supplementary material for: Modeling glioblastoma heterogeneity as a dynamic network of cell states
Source: Mol Syst Biol. 2021 Sep 16;17(9):e10105. doi: 10.15252/msb.202010105 (PMC8444284; doi:10.15252/msb.202010105)
Supplement: Supplementary file 6 — Source Data for Figure 5 [file MSB-17-e10105-s004.zip › Figure5A_sourcedata/GSEA_3071/nosigdb_stateIV.GseaPreranked.1622795164626/RICHARDS_NOWAKOWSKI_SCIENCE_2017_NIN3_UPREG.html]

Details for gene set RICHARDS\_NOWAKOWSKI\_SCIENCE\_2017\_NIN3\_UPREG[GSEA]

|  || Dataset | state53071 |
| Phenotype | NoPhenotypeAvailable |
| Upregulated in class | na\_pos |
| GeneSet | RICHARDS\_NOWAKOWSKI\_SCIENCE\_2017\_NIN3\_UPREG |
| Enrichment Score (ES) | 0.5384387 |
| Normalized Enrichment Score (NES) | 2.9738064 |
| Nominal p-value | 0.0 |
| FDR q-value | 0.0 |
| FWER p-Value | 0.0 |
Table: GSEA Results Summary

  

Fig 1: Enrichment plot: RICHARDS\_NOWAKOWSKI\_SCIENCE\_2017\_NIN3\_UPREG      
 Profile of the Running ES Score & Positions of GeneSet Members on the Rank Ordered List

  

| PROBE | GENE SYMBOL | GENE\_TITLE | RANK IN GENE LIST | RANK METRIC SCORE | RUNNING ES | CORE ENRICHMENT || 1 | DCX |  |  | 4 | 2.752 | 0.0317 | Yes |
| 2 | SNTG1 |  |  | 8 | 2.543 | 0.0612 | Yes |
| 3 | DLX5 |  |  | 17 | 2.283 | 0.0866 | Yes |
| 4 | PLXNA4 |  |  | 20 | 2.236 | 0.1127 | Yes |
| 5 | DLX2 |  |  | 50 | 1.858 | 0.1290 | Yes |
| 6 | SOX11 |  |  | 56 | 1.811 | 0.1495 | Yes |
| 7 | DLX1 |  |  | 62 | 1.768 | 0.1694 | Yes |
| 8 | DCLK2 |  |  | 74 | 1.704 | 0.1874 | Yes |
| 9 | DPYSL3 |  |  | 75 | 1.691 | 0.2074 | Yes |
| 10 | TCF4 |  |  | 96 | 1.551 | 0.2218 | Yes |
| 11 | NEDD4L |  |  | 100 | 1.535 | 0.2394 | Yes |
| 12 | LSAMP |  |  | 130 | 1.454 | 0.2510 | Yes |
| 13 | CCDC88A |  |  | 135 | 1.429 | 0.2671 | Yes |
| 14 | CHD7 |  |  | 144 | 1.386 | 0.2819 | Yes |
| 15 | DLX6 |  |  | 145 | 1.384 | 0.2983 | Yes |
| 16 | BCL11A |  |  | 154 | 1.367 | 0.3129 | Yes |
| 17 | BCL11B |  |  | 163 | 1.343 | 0.3272 | Yes |
| 18 | NRXN3 |  |  | 170 | 1.321 | 0.3417 | Yes |
| 19 | SP9 |  |  | 189 | 1.281 | 0.3533 | Yes |
| 20 | GAD1 |  |  | 190 | 1.281 | 0.3685 | Yes |
| 21 | SLAIN1 |  |  | 203 | 1.257 | 0.3810 | Yes |
| 22 | SOX6 |  |  | 207 | 1.244 | 0.3951 | Yes |
| 23 | ASAP1 |  |  | 211 | 1.231 | 0.4091 | Yes |
| 24 | CADPS |  |  | 220 | 1.205 | 0.4218 | Yes |
| 25 | SATB1 |  |  | 222 | 1.203 | 0.4358 | Yes |
| 26 | KCNQ1OT1 |  |  | 246 | 1.159 | 0.4451 | Yes |
| 27 | RBP1 |  |  | 254 | 1.136 | 0.4571 | Yes |
| 28 | FBXW7 |  |  | 260 | 1.129 | 0.4695 | Yes |
| 29 | PFN2 |  |  | 270 | 1.107 | 0.4808 | Yes |
| 30 | ARL4D |  |  | 295 | 1.039 | 0.4885 | Yes |
| 31 | PDZRN4 |  |  | 318 | 1.015 | 0.4962 | Yes |
| 32 | SOX4 |  |  | 377 | 0.928 | 0.4960 | Yes |
| 33 | PDZRN3 |  |  | 405 | 0.904 | 0.5015 | Yes |
| 34 | KLHL13 |  |  | 412 | 0.893 | 0.5109 | Yes |
| 35 | ARX |  |  | 415 | 0.891 | 0.5210 | Yes |
| 36 | PNRC1 |  |  | 481 | 0.830 | 0.5183 | Yes |
| 37 | FOS |  |  | 530 | 0.786 | 0.5184 | Yes |
| 38 | HIP1 |  |  | 563 | 0.753 | 0.5211 | Yes |
| 39 | CDKN1C |  |  | 565 | 0.751 | 0.5298 | Yes |
| 40 | ST8SIA4 |  |  | 567 | 0.751 | 0.5384 | Yes |
| 41 | BMPR2 |  |  | 677 | 0.673 | 0.5254 | No |
| 42 | RAB5B |  |  | 704 | 0.658 | 0.5281 | No |
| 43 | MUM1 |  |  | 706 | 0.657 | 0.5357 | No |
| 44 | FIGN |  |  | 853 | 0.578 | 0.5144 | No |
| 45 | PPP4R2 |  |  | 905 | 0.558 | 0.5112 | No |
| 46 | SLC25A36 |  |  | 1061 | 0.503 | 0.4872 | No |
| 47 | SYNE2 |  |  | 1094 | 0.493 | 0.4869 | No |
| 48 | HMBOX1 |  |  | 1113 | 0.488 | 0.4892 | No |
| 49 | IGF1R |  |  | 1140 | 0.482 | 0.4899 | No |
| 50 | TP53INP1 |  |  | 1174 | 0.473 | 0.4891 | No |
| 51 | GPBP1 |  |  | 1244 | 0.453 | 0.4811 | No |
| 52 | SERINC5 |  |  | 1383 | 0.421 | 0.4595 | No |
| 53 | KIZ |  |  | 1402 | 0.416 | 0.4609 | No |
| 54 | DLL1 |  |  | 1463 | 0.399 | 0.4541 | No |
| 55 | MAPK10 |  |  | 1471 | 0.398 | 0.4574 | No |
| 56 | GRIA4 |  |  | 1545 | 0.379 | 0.4478 | No |
| 57 | MOB3B |  |  | 1552 | 0.377 | 0.4511 | No |
| 58 | SGCB |  |  | 1593 | 0.369 | 0.4478 | No |
| 59 | NDUFAB1 |  |  | 1628 | 0.363 | 0.4455 | No |
| 60 | MTRNR2L1 |  |  | 1697 | 0.350 | 0.4365 | No |
| 61 | HOOK3 |  |  | 1751 | 0.339 | 0.4303 | No |
| 62 | ERBB4 |  |  | 1843 | 0.325 | 0.4166 | No |
| 63 | SKIDA1 |  |  | 1869 | 0.318 | 0.4156 | No |
| 64 | MFN1 |  |  | 1891 | 0.315 | 0.4152 | No |
| 65 | RBM4B |  |  | 1935 | 0.307 | 0.4106 | No |
| 66 | EPHA4 |  |  | 2032 | 0.292 | 0.3955 | No |
| 67 | TET2 |  |  | 2082 | 0.286 | 0.3894 | No |
| 68 | MAF |  |  | 2206 | 0.267 | 0.3689 | No |
| 69 | C2orf68 |  |  | 2213 | 0.266 | 0.3709 | No |
| 70 | CNOT2 |  |  | 2256 | 0.261 | 0.3659 | No |
| 71 | SMARCA5 |  |  | 2322 | 0.251 | 0.3563 | No |
| 72 | SCP2 |  |  | 2368 | -0.255 | 0.3506 | No |
| 73 | RASA1 |  |  | 2437 | -0.262 | 0.3406 | No |
| 74 | DUSP10 |  |  | 2539 | -0.274 | 0.3244 | No |
| 75 | RHOB |  |  | 2638 | -0.288 | 0.3089 | No |
| 76 | ARPC3 |  |  | 2702 | -0.295 | 0.3002 | No |
| 77 | RAP2B |  |  | 2777 | -0.303 | 0.2895 | No |
| 78 | CHORDC1 |  |  | 3133 | -0.351 | 0.2252 | No |
| 79 | PDHA1 |  |  | 3244 | -0.367 | 0.2084 | No |
| 80 | RNF149 |  |  | 3258 | -0.369 | 0.2102 | No |
| 81 | HSPA1A |  |  | 3371 | -0.386 | 0.1932 | No |
| 82 | ST7 |  |  | 3378 | -0.387 | 0.1966 | No |
| 83 | HSPA1B |  |  | 3393 | -0.390 | 0.1985 | No |
| 84 | STIP1 |  |  | 3427 | -0.395 | 0.1968 | No |
| 85 | RPL21 |  |  | 3429 | -0.395 | 0.2013 | No |
| 86 | MYO1B |  |  | 3431 | -0.396 | 0.2058 | No |
| 87 | ACTB |  |  | 3512 | -0.412 | 0.1952 | No |
| 88 | HSP90AB1 |  |  | 3618 | -0.432 | 0.1801 | No |
| 89 | DIMT1 |  |  | 3915 | -0.490 | 0.1288 | No |
| 90 | GFPT1 |  |  | 4014 | -0.516 | 0.1160 | No |
| 91 | HSP90AA1 |  |  | 4024 | -0.519 | 0.1204 | No |
| 92 | ADAMTS5 |  |  | 4047 | -0.525 | 0.1224 | No |
| 93 | CACYBP |  |  | 4109 | -0.542 | 0.1170 | No |
| 94 | CITED2 |  |  | 4303 | -0.598 | 0.0869 | No |
| 95 | POLE3 |  |  | 4336 | -0.610 | 0.0879 | No |
| 96 | RND3 |  |  | 4402 | -0.635 | 0.0829 | No |
| 97 | CCT4 |  |  | 4613 | -0.740 | 0.0511 | No |
| 98 | HSPB1 |  |  | 4701 | -0.797 | 0.0438 | No |
| 99 | PDE4DIP |  |  | 4715 | -0.804 | 0.0508 | No |
| 100 | EIF1 |  |  | 4822 | -0.889 | 0.0408 | No |
| 101 | ACTG1 |  |  | 4882 | -0.947 | 0.0406 | No |
| 102 | SH3BGRL3 |  |  | 4911 | -0.978 | 0.0468 | No |
| 103 | KLF6 |  |  | 4953 | -1.024 | 0.0510 | No |
| 104 | IGF2BP1 |  |  | 5036 | -1.166 | 0.0490 | No |
Table: GSEA details [plain text format]

  

Fig 2: RICHARDS\_NOWAKOWSKI\_SCIENCE\_2017\_NIN3\_UPREG: Random ES distribution      
 Gene set null distribution of ES for **RICHARDS\_NOWAKOWSKI\_SCIENCE\_2017\_NIN3\_UPREG**
